# Research note: Turkey (*Meleagris gallopavo*) semen CD14^+^ macrophages – management, key features, and classification – a pilot study with optimized protocols^☆^

**DOI:** 10.1016/j.psj.2025.105397

**Published:** 2025-06-07

**Authors:** Ewa M. Drzewiecka, Anna Szóstek-Mioduchowska, Joanna Wiśniewska, Ewa Liszewska, Mariola Słowińska

**Affiliations:** a Team of Gamete Biology, Institute of Animal Reproduction and Food Research, Polish Academy of Sciences in Olsztyn, Trylińskiego 18, 10-683 Olsztyn, Poland; b Team of Molecular Basis of Equine Reproduction, Institute of Animal Reproduction and Food Research, Polish Academy of Sciences in Olsztyn, Trylińskiego 18, 10-683 Olsztyn, Poland; c Laboratory of Cell Analysis, Institute of Animal Reproduction and Food Research, Polish Academy of Sciences in Olsztyn, Trylińskiego 18, 10-683 Olsztyn, Poland

**Keywords:** Turkey, Semen macrophages, CD14^+^Mф, FACS, IF staining

## Abstract

Semen macrophages (**Mф**) play a role in mediating innate immune responses and supporting adaptive immunity within the male reproductive tract. Knowledge about the semen Mф in avian species remains limited. This study aimed to isolate turkey semen Mф and optimize protocols for their characterization based on specific Mф markers, specifically cluster of differentiation 14 (**CD14**), inducible nitric oxide synthase (**iNOS**), and interleukin 10 (**IL10**), for further classification into CD14^+^ type 1 macrophages (CD14^+^iNOS^+^, akin **CD14^+^Mф1**) and CD14^+^ type 2 macrophages (CD14^+^IL10^+^, akin **CD14^+^Mф2**) subpopulations. In the current study, immunofluorescence (**IF**) staining and fluorescence-activated cell sorting (**FACS**) were employed to identify these markers, and the morphological features of CD14^+^Mф were examined. Our results demonstrated that turkey semen CD14^+^Mф are spherical, may contain one or more nuclei, and often exhibit large vacuoles. Based on FACS results, CD14^+^iNOS^+^(akin CD14^+^Mф1) accounted for 48.5 ± 12.45 % of CD14^+^ cells, whereas CD14^+^IL10^+^ (akin CD14^+^Mф2) represented 42.48 ± 14.62 % of them. This study provides a foundational framework for future investigations into the functional roles of turkey CD14^+^Mф in semen.

## Introduction

Semen macrophages (**Mф**) mediate innate and support adaptive immune responses in the reproductive tract (Chen and Zheng, 2021). In humans, the presence of Mф in semen is considered physiological and may contribute to protecting sperm from foreign antigens (Chen and Zheng, 2021). However, an increased concentration of Mф in semen might be linked to impaired semen quality and a greater incidence of infertility (Chen and Zheng, 2021). Knowledge about the properties and functions of semen Mф in avian species remains limited.

To date, research on turkey semen Mф has primarily relied on electron microscopy imaging, with a demonstration of some characteristics, such as nonspecific phagocytosis and esterase activity (Marquez and Ogasawara, 1975; Korn et al., 1998). However, none of the classic Mф markers have been assessed in turkey semen Mф so far (Marquez and Ogasawara, 1975; Korn et al., 1998). In studies on semen Mф, it is essential to define these cells based on the expression of the marker that does not appear on other cells present in semen. One such is a cluster of differentiation 14 (**CD14**) (Murray et al., 2014; Chen and Zheng, 2021). Noteworthy, CD14^+^Mф are generally classified into pro-inflammatory (CD14^+^ type 1 macrophages, akin **CD14^+^Mф1**) and anti-inflammatory (CD14^+^ type 2 macrophages, akin **CD14^+^Mф2**) subpopulations, which may switch phenotypes depending on the properties of their microenvironment (Murray et al., 2014; Yunna et al., 2020; Chen and Zheng, 2021). Numerous markers are used to define these subpopulations, including inducible nitric oxide synthase (**iNOS**) and interleukin 10 (**IL10**) (Murray et al., 2014; Yunna et al., 2020). Here, to enhance clarity, we designate turkey semen CD14^+^iNOS^+^ cells as CD14^+^Mф1, and turkey semen CD14^+^IL10^+^ cells as CD14^+^Mф2.

This study aimed to isolate turkey semen Mф and optimize protocols for their characterization based on specific Mф markers, facilitating their classification into CD14^+^Mф1 and CD14^+^Mф2 subpopulations using immunofluorescence (**IF**) staining and fluorescence-activated cell sorting (**FACS**). The present study may serve as a foundation for future investigations into the functional roles of turkey semen CD14^+^Mф in regulating immune processes that may be associated with semen quality in this species.

## Material and methods

### Ethical statement

All experiments involving animals were conducted in accordance with national guidelines for agricultural animal care and veterinary practice, following EU Directive 2010/63/UE.

### Animals and sample collection

Turkey toms BUT6 (Aviagen Turkeys, Tattenhall, Cheshire, Great Britain) were maintained under standard husbandry conditions at the parent stock farm of Zdrowy Drob Ltd. in Frednowy, Indykpol, Drosed Group (Poland). Feed and water were provided *ad libitum*. Animals were photostimulated at 26 weeks of age (14 h light to 10 h darkness) and produced semen by 30 weeks old. The sampling time was chosen within the reproductive season. All experiments were conducted on semen collected from healthy toms by abdominal massage (Burrows and Quinn, 1937).

### Semen macrophage (Mф) isolation

For transportation, individual semen samples were suspended in 4 mL of transport medium [Hanks Balanced Salt Solution (H1387-10 × 1 L, Merck, USA) supplemented with glucose (4,500 mg/L; G7528, Sigma Aldrich, Germany), and 1 % antibiotic-antimycotic solution (**AA**; A5955-100ML, Merck, USA)]. Diluted semen samples were transported on ice.

In the laboratory, individual semen samples were supplemented with a fresh portion of the transport medium up to 10 mL of sample volume, and washed by centrifugation at 600 × *g*, 12 min, 4 °C. Afterward, the supernatant was discarded, and centrifuged cells were designated for the cold aggregation protocol (Shen et al., 1986; Santos et al., 2001; Chometon et al., 2020). The cold aggregation protocol at this stage was designed to induce aggregation and sedimentation of Mф, and thereby facilitate their separation, particularly those that form multinucleated giant cells – a subset of activated Mф possibly present in turkey semen – that could not be adequately separated using gradient-based methods (Ahmadzadeh et al., 2022). To do so, centrifuged cells were gently mixed with 8 mL of preheated to 38 °C high-glucose Dulbecco′s Modified Eagle′s Medium (**DMEM**; D6429-500ML, Merck, USA) supplemented with 20 % Fetal Bovine Serum (**FBS**, 10270106, Thermo Fisher Scientific, USA) and 1 % of AA. Samples were gently agitated in a horizontal position for 30 min at 4 °C. Next, samples were put in a vertical position in an ice bath for 8 min, allowing for sedimentation of aggregated Mф to the bottom of the tube. These cells were kept on ice until further use, and the supernatant was further processed to increase Mф yield with possibly remaining mononucleated Mф fraction.

For this, the samples of the supernatant obtained after the cold aggregation, possibly containing remaining mononucleated Mф fraction, were washed in phosphate-buffered saline (**PBS**) with 3 % AA, and centrifuged at 600 × *g*, 12 min, 4 °C. After centrifugation, the supernatant was discarded, and centrifuged cells were gently mixed up with PBS with 3 % AA up to 6 mL of the final volume, overlaid on 4 mL of chilled Ficoll-Plaque Plus medium (GE17-1440-02, Merck, USA), and centrifuged at 3,000 × *g*, 20 min, 10 °C. Next, the buffy coat containing mononucleated cells was aspirated and combined with cells obtained with the first step of the cold aggregation protocol for further washing in PBS with 3 % AA, and centrifugation at 600 × *g*, 12 min, 4 °C. The isolated Mф from individual samples were suspended in 1 mL of DMEM with 20 % FBS and 1 % AA and counted and tested for viability after trypan-blue staining (T8154-20ML, Merck, USA) in Bürker’s chamber.

The obtained semen Mф were subjected to cell culture or cryopreservation. Cells designated for culture were used further for cell viability test, phagocytosis assay, and analysis of the expression of CD14, iNOS, and IL10 markers with IF staining. Cells designated for cryopreservation were suspended in FBS with 10 % dimethyl sulfoxide (**DMSO**, D8418-50ML, Merck, Germany) in a concentration of 6 × 10^7^ cells/ mL and stored at −80 °C until further use for FACS based on the expression of CD14, iNOS, and IL10 markers.

### Cell culture

Based on our preliminary data on turkey toms (*n* = 8) with good semen quality (> 60 % motile sperm, <20 mg/mL protein in seminal plasma), with semen volume ranging between 0.135 and 0.335 mL, the mean recovery of semen Mф from individual tome was 546,241 cells/ejaculate (range: 166,660 - 1,111,111 cells/ejaculate, depending on semen volume). Thus, for cell culture, Mф collected from individual donors were pooled to ensure a final concentration of 0.5 × 10^6^ viable (>95 %) Mф in 0.2 mL culture medium [DMEM (D6429-500ML, Merck, USA) supplemented with 20 % FBS (10270106, Thermo Fisher Scientific, USA) and 1 % AA]. Cells from one pool were considered as one biological replicate. Cells were seeded on CELLview cell culture slides (543078, Greiner Bio-One, Austria), and cultured at 41 °C, in the humidified atmosphere of 5 % CO_2_. After 1 h, floating cells were aspirated, a fresh medium was added, and the culture was held for the next 24 h. The next day, cells were washed in warmed PBS with 3 % AA and subjected to viability test (*n* = 2), phagocytosis assay (*n* = 2), or immunofluorescent staining (*n* = 15).

### Cell viability and phagocytosis assay

One-day-old cell cultures of semen Mф were tested for viability using an In Vitro Toxicology Assay Kit, MTT-based (TOX1-1KT, Sigma Aldrich, Germany), following the manufacturer’s protocol. The phagocytosis properties were tested using the Phagocytosis Assay Kit (Red Zymosan) (AB234054, Abcam, United Kingdom). Zymosan particles were added to a culture medium 24 h after seeding, and phagocytosis was observed in real-time at 41 °C, in the humified atmosphere of 5 % CO_2_ using fluorescence microscopy with an Axio Observer. Z1/7 fluorescence microscope (Carl Zeiss, Inc., Oberkochen, Germany) equipped with ZEN 2.3 blue edition software (Carl Zeiss).

### Determination of CD14, iNOS, and IL10 expression in turkey semen Mф – IF staining

One-day-old cell cultures prepared as above were washed in warmed PBS with 3 % AA, and fixed with warmed 4 % formaldehyde (432173111, POCh, Poland) for 8 min at room temperature (**RT**). Cells were then washed with chilled PBS and subjected to IF staining. First, the area of cell growth was limited with a Pap pen for immunostaining (Z672548-1EA, Merck, Germany). Next, cells were permeabilized with 0.25 % Triton X100 (T8787-100ML, Merck, Germany) in PBS for 10 min at RT, triple-washed with PBS, and incubated in a blocking medium consisting of 1 % bovine serum albumin (**BSA**, A1470-100 G, Merck, Germany), 22.52 mg/mL glycine (527560117, POCh, Poland), 0.1 % Tween 20 (P-2287, Sigma Aldrich, Germany), and 10 % DMSO in PBS for 30 min at RT. Next, cells were triple-washed with PBS and covered with primary mouse anti-CD14 antibodies (orb434888, Biorbyt, United Kingdom, 1:50 in PBS). After 60 min at RT, cells were triple-washed with PBS, and covered with a mix of primary rabbit anti-iNOS antibodies conjugated with R-PE (bs-2072R-PE, Bioss Antibodies, USA, 1:50), primary goat anti-IL10 antibodies (orb379727, Biorbyt, United Kingdom, 1:50) conjugated with Alexa Fluor 647 (Alexa Fluor 647 Conjugation kit (Fast) - Lightning Ling #ab269823, Abcam, United Kingdom, 1 mg/mL anti-IL10 per 100 ug kit) and secondary Alexa Fluor 488 Goat Anti-Mouse IgG (A-11029, ThermoFisher Scientific, USA, 1:200). After the next 60 min at RT, cells were triple-washed with PBS, cells were co-stained with 20 μg/mL 4′,6-diamidino-2-phenylindole (**DAPI**) solution (Sigma-Aldrich, Germany) for 5 min at RT, triple-washed with PBS, and finally mounted using Fluoreoshield with DAPI (F6057-20ML, Merck, Germany). In parallel to the analyzed samples, a control sample consisting of non-stained cells (negative control) was prepared. A fluorescence signal was detected confocal Laser Scanning Microscope (LSM800; Carl-Zeiss, Germany) equipped with an Airyscan super-resolution module supported by Zen Blue Pro 2.6 software (Carl-Zeiss; Germany). A total of 35 randomly selected CD14^+^ cells were measured for size (µm).

### Identification of CD14, iNOS, and IL10 positive cell population – FACS

For FACS, the same reagents as in IF staining were used until otherwise stated. Only non-pooled samples from individual donors were used. Before staining, cells (*n* = 4) were thawed at 37 °C, and washed by centrifugation in PBS at 600 × *g*, 12 min, 4 °C. Next, cells were fixed in 4 % formaldehyde for 8 min at RT, and washed with centrifugation as above. Cells were then mixed up with 0.25 % Triton X100 in PBS for 10 min at RT for permeabilization. After subsequent washing in PBS and centrifugation, cells were suspended in a blocking medium consisting of 1 % BSA, 22.52 mg/mL glycine, 0.1 % Tween 20 in PBS, and incubated for 30 min at 4 °C. Afterwards, cells were washed in PBS, and centrifuged, and used for two-step staining. First, cells were suspended in primary mouse anti-CD14 antibodies (orb434888, Biorbyt, United Kingdom) conjugated with FITC (FITC Conjugation Kit (Fast) - Lightning-Link #ab1888285, Abcam, United Kingdom, 0.75 mg/mL mouse anti-CD14 in PBS per 100 μg kit) diluted 1:4 in PBS. After 30 min incubation at RT, primary rabbit anti-iNOS antibodies conjugated with R-PE (bs-2072R-PE, Bioss Antibodies, USA) and primary goat anti-IL10 antibodies (orb379727, Biorbyt, United Kingdom) conjugated with Alexa Fluor 647 (Alexa Fluor 647 Conjugation kit (Fast) - Lightning Ling #ab269823, Abcam, United Kingdom, 1 mg/mL anti-IL10 per 100 ug kit) were added in final dilution 1:19, and incubated for next 60 min at RT. Finally, cells were washed, centrifuged, and suspended in PBS for signal detection in BD FACSAria II Cell Sorter, Beckton Dickinson, and analyzed using BD FCSDiva Software v6.1.3, Beckton Dickinson. The total number of cells analyzed for each sample was 10000. The cells were analyzed using multi-wavelength analysis (green [530/30 nm], orange [585/42 nm], and red [660/20 nm]) after excitation with 488-nm and 633-nm light. Fluorescence compensation was performed prior to cytometric analysis to correct for the spectral overlap of fluorochrome emission. In parallel to the analyzed samples, a control sample consisting of non-stained semen Mф was prepared. The percentage of obtained CD14^+^Mф, CD14^+^iNOS^+^ (akin CD14^+^Mф1), and CD14^+^IL10^+^ (akin CD14^+^Mф2) cells, as well as the CD14^+^Mф1/CD14^+^Mф2 ratio in the analyzed samples was assessed. The data are presented as mean ± standard deviation. The percentage of CD14^+^Mф1 and CD14^+^Mф2 cells was compared with a t-test.

## Results and discussion

The current study, for the first time, shows molecular features of turkey semen Mф using IF staining and FACS. The currently used isolation protocol, based on Korn et al. (1998), with modifications including the cold-aggregation step (Shen et al., 1986; Santos et al., 2001; Chometon et al., 2020), allows for obtaining semen Mф of acceptable vitality (> 95 % live cells) from individual donors. Previously, only pooled semen samples from several donors were used for Mф isolation (Korn et al., 1998). This approach impeded further experiments aiming to characterize semen Mф in individual birds for, i.a., the determination of their impact on semen quality in turkeys. From now on, such studies are accessible, especially ones that utilize FACS, and may help in designing experiments allowing for full characterization of turkey semen Mф to understand and resolve immune-dependent fertility issues in this species. However, for methodologies involving cell culture, pooling of samples is generally necessary, since the cell yield from a single donor is often insufficient to initiate a culture.

**Fig. 1:**
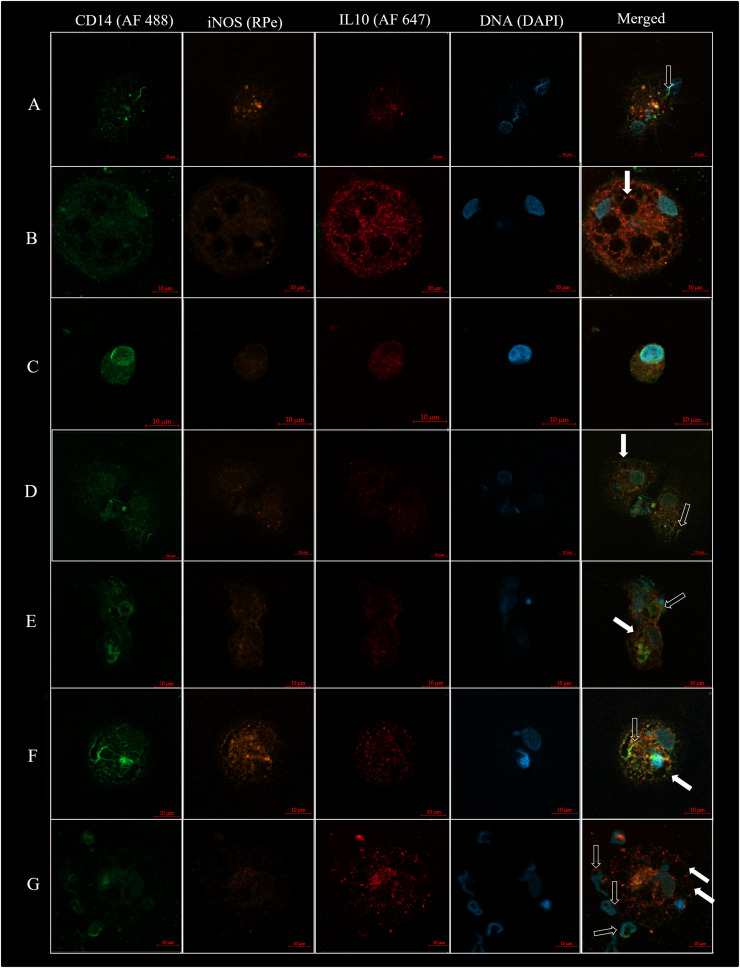
The visualization of turkey macrophages using immunofluorescence (IF) staining. **A.** A representative turkey semen CD14^+^ type 1 macrophages (CD14^+^iNOS^+^, akin CD14^+^Mф1); **B.** A representative turkey semen CD14^+^ type 2 macrophages (CD14^+^IL10^+^, akin CD14^+^Mф2); **C.** A representative small mononucleated semen CD14^+^Mф with an absence of vacuoles; **d-F.** Hypothetical process of multinucleated giant cell formation from two mononucleated small semen CD14^+^Mф; **G.** Multinucleated giant semen CD14^+^Mф with internalized sperm cells. Full arrows indicate vacuoles, and empty arrows indicate selected sperm. Images were captured using a confocal Laser Scanning Microscope (LSM800; Carl-Zeiss, Germany) equipped with an Airyscan super-resolution module (40 × /1.2 NA W c-apochromat) supported by Zen Blue Pro 2.6 software (Carl-Zeiss; Germany).

Previously, turkey semen Mф were visualized using electron microscopy (Marquez and Ogasawara, 1975). Noteworthy, early studies on turkey semen Mф have not assessed any of the Mф markers (Marquez and Ogasawara, 1975; Korn et al., 1998). Used in the current study IF staining confirmed, for the first time, that turkey semen Mф are CD14^+^ and may possess Mф1 and Mф2 features, i.e. expression of iNOS or/and IL10 markers, respectively (Fig. 1). Notably, determining a larger spectrum of Mф1 and Mф2 markers might be needed in further in-depth studies aiming for full characterization of turkey semen Mф; however, our current report might be recognized as a firm starting point for future research.

Based on current qualitative analyses, we confirmed that turkey semen CD14^+^Mф are rather spherical, mean diameter (**ø**) = 32.42 µm, min *ø* = 12 µm, max *ø* = 78 µm (Fig. 1). These cells might contain one (Fig. 1A, C-E) or more (Fig. 1B, F, G) nuclei. This is in line with the literature data showing that turkey semen CD14^+^Mф may form multinucleated giant cells (Ahmadzadeh et al., 2022). For this reason, simple density-gradient centrifugation might be insufficient to obtain all semen CD14^+^Mф subpopulations for their variety of morphological forms. Thus, we believe that adding a cold-aggregation as the first step of semen Mф isolation is gainful in obtaining the fraction enriched with multinucleated giant cells, for full characterization of their population in further research.

**Fig. 2:**
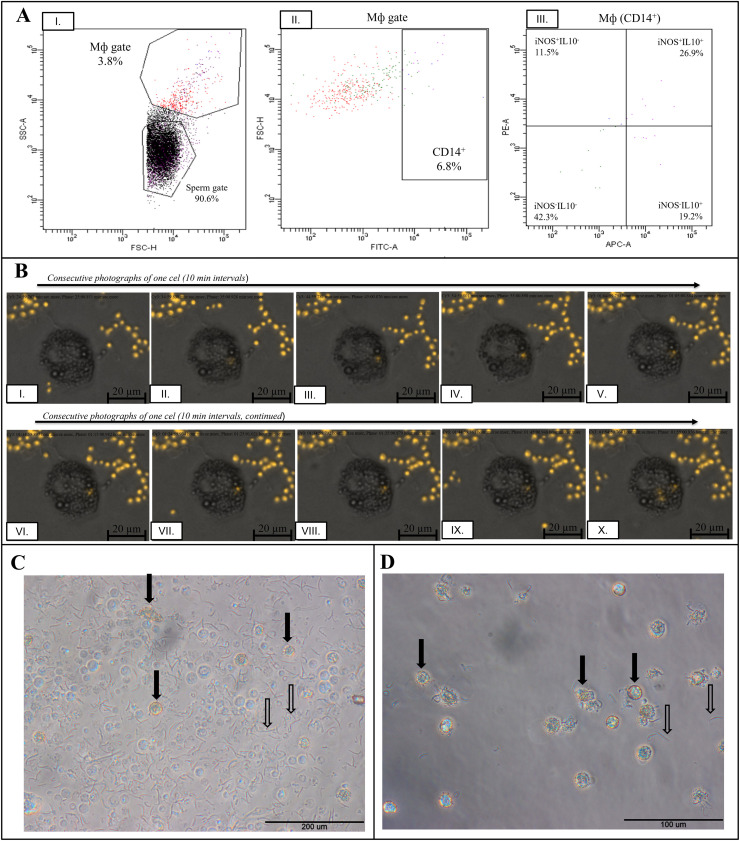
**A.** Turkey semen CD14^+^Mф measured by flow cytometry I-III: The representative cytogrammes show the gating strategy for identifying the different CD14^+^Mф subpopulations. **B.** I-X: Consecutive photographs (10-min intervals) of turkey semen Mф phagocyting zymosan particle imaged using fluorescence microscopy with an Axio Observer. Z1/7 fluorescence microscope (Carl Zeiss, Inc., Oberkochen, Germany) equipped with ZEN 2.3 blue edition software (Carl Zeiss). **C.** Turkey semen Mф 24 h after seeding (magnification 200 ×), and **D.** after culture washing (magnification 400 ×). The high incidence of sperm in the cell pool decreases when cultured in high-glucose DMEM and after cell washing. Full arrows indicate selected Mф and empty arrows indicate sperm.

Remarkably, characterized in the current study, semen CD14^+^Mф may show iNOS or IL10 expression (Fig. 1A, B, respectively), allowing for their classification to CD14^+^Mф1 and CD14^+^Mф2. Using a FACS analysis, we demonstrated that among the analyzed individuals, only 4.9 ± 1.45 % of cells initially gated as Mф were CD14 positive (Fig. 2A). Among these CD14^+^ cells (CD14^+^Mф), 48.5 ± 12.45 % were CD14^+^iNOS^+^(akin CD14^+^Mф1), whereas CD14^+^IL10^+^ (akin CD14^+^Mф2) represented 42.48 ± 14.62 %. This gives the CD14^+^Mф1/CD14^+^Mф2 ratio equal to 1.29. No statistically significant differences (*P* > 0.05) were found between the percentage of CD14^+^Mф1 and CD14^+^Mф2 cells within the studied individuals. Further studies are necessary to investigate whether the abundance of CD14^+^Mф and CD14^+^Mф2/CD14^+^Mф2 ratio vary in relation to semen quality.

Based on the currently obtained data, we noted that many cells possess characteristic vacuoles (Figs. 1A, B, E-G and 2B), confirming their activated state. In the present study, we observed the buoyant phenotype of turkey semen Mф, leading to poor adhesion while cultured. This feature of turkey semen Mф was also observed previously (Korn et al., 1998). This raises further challenges to establish turkey semen CD14^+^Mф cell culture. Future research on turkey semen CD14^+^Mф may probably require using suspension rather than adherent cell culture.

Fluorescent staining visualized sperm cells inside macrophages (Fig 1A, F, G), highlighting their potential role in eliminating abnormal or low-quality sperm. This might be an interesting idea for further studies. Remarkably, the phagocytosis activity of turkey semen Mф against sperm cells was determined firstly with electron microscopy (Marquez and Ogasawara, 1975), and for this, they were called spermiophages (Korn et al., 1998). Phagocytosis is an evolutionarily conserved function of the Mф, however, it is not specific to all available Mф subpopulations (Murray et al., 2014). Following Korn et al. (1998), phagocytic properties of turkey semen Mф against zymosan were confirmed only in 20 % of the tested cells. Our results confirm that in some cells, phagocytosis against zymosan particles was observed (Fig. 2B). Additional experiments to provide an estimation of the phagocytosis activity in semen CD14^+^Mф1 and CD14^+^Mф2, separated based on their molecular features, would be advantageous in further studies.

It is worth highlighting that the main problem in turkey semen Mф isolation was the very low yield from individual donors, generally not exceeding 1 × 10^6^ cells/ejaculate. For FACS analyses, this problem might be overcame by resigning from complete sperm removal from the final sample, especially since it forms a separated population in cytogrammes (Fig. 2A). However, too high concentration of sperm in samples intended for FACS can impede the separation of the Mф population and contribute to a high background signal, particularly in the FITC channel, thereby disrupting accurate signal detection. Notably, even samples that underwent Mф isolation protocol may give a high background. Nevertheless, analyzing beforehand cryopreserved semen Mф in FBS with 10 % DMSO instead of freshly isolated cells eliminates this problem (data not shown).

For proteomic or transcriptomic analyses, careful separation of the desired CD14^+^Mф population is needed. Removal of sperm cells from Mф fraction in many cases might be achieved by additional centrifugation of the obtained cells in the medium, e.g., 600 × *g*, 12 min, 4 °C, and 350 × *g*, 20 min, 4 °C, or optionally additional density-gradient centrifugation (data not shown). After seeding Mф in high-glucose DMEM, residual sperm cells can be effectively removed through routine medium changes (Fig. 2C, D). Nevertheless, still a possibility of contamination with other leukocytes is possible. Thus, for methods where the undeniable purity of CD14^+^ cells is needed, we recommend using sorters to enable CD14^+^ cells separation. However, in this case, starting a culture might be challenging for the low yield of cells that might be isolated from an individual donor. If so, there is a need to pool the CD14^+^Mф from several individual donors, e.g., based on the same age, and semen quality parameters like sperm motility and viability, seminal plasma protein concentration, and others.

## Conflict of interest

The authors declare no conflicts to disclose.

During the preparation of this work, the authors used Grammarly and ChatGPT to correct the language. After using these tools, the authors reviewed and edited the content as needed and take full responsibility for the content of the publication.

## References

[bib0001] Ahmadzadeh K., Vanoppen M., Rose C.D., Matthys P., Wouters C.H. (2022). Multinucleated giant cells: current insights in phenotype. Biol. Act. Mech. Form. Front. Cell Dev. Biol..

[bib0002] Burrows W.H., Quinn J.P. (1937). The collection of spermatozoa from the domestic fowl and turkey. Poult. Sci..

[bib0003] Chen G., Zheng B. (2021). Effect of macrophages in semen on sperm quality. Reprod. Biol. Endocrinol..

[bib0004] Chometon T.Q., de Silva Siqueira M.., Sant´anna J.C., Almeida M.R., Gandini M., Martins de Almeida Nogueira A.C., Antas P.R.Z., Martins de Almeida A.C. (2020). A protocol for rapid monocyte isolation and generation of singular human monocyte-derived dendritic cells. PLoS. One.

[bib0005] Korn N., Barnes D.A., Williams C.A., Thurston R.J. (1998). Physiology and reproduction characterization of turkey spermiophages with regard to traits common to macrophages. Poult. Sci..

[bib0006] Marquez B.J., Ogasawara F.X. (1975). Scanning electron microscope studies of turkey semen. Poult. Sci...

[bib0007] Murray P.J., Allen J..E., Biswas S.K., Fisher E.A., Gilroy D.W., Goerdt S., Gordon S., Hamilton J.A., Ivashkiv L.B., Lawrence T., Locati M., Mantovani A., Martinez F.O., Mege J.L., Mosser D.M., Natoli G., Saeij J.P., Schultze J.L., Shirey K.A., Sica A., Suttles J., Udalova I., vanGinderachter J.A., Vogel S.N., Wynn T.A. (2014). Macrophage Act. Polariz.: Nomencl. Exp. Guidel. Immun..

[bib0008] Santos D.O., Santos S..L., Esquenazi D., Nery J.A., Defruyt M., Lorre K., Van Heuverswyn H. (2001). Evaluation of B7-1 (CD80) and B7-2 (CD86) costimulatory molecules and dendritic cells on the immune response in leprosy. Jpn. J. Lepr..

[bib0009] Shen L.I., Guyre P..M., Ball E.D., Fanger M.W. (1986). Glucocorticoid enhances gamma interferon effects on human monocyte antigen expression and ADCC. Clin. Exp. Immunol..

[bib0010] Yunna C., Mengru H., Lei W., Weidong C. (2020). Macrophage M1/M2 polarization. Eur. J. Pharmacol..

